# How pragmatic or explanatory is the randomized, controlled trial? The application and enhancement of the PRECIS tool to the evaluation of a smoking cessation trial

**DOI:** 10.1186/1471-2288-12-101

**Published:** 2012-07-23

**Authors:** Peter Selby, Gerald Brosky, Paul I Oh, Vincent Raymond, Suzanne Ranger

**Affiliations:** 1 Addictions Program, Centre for Addiction and Mental Health, 100 Stokes St., 33 Russell Street, Toronto, ON M6J 1H4, Canada; 2 Departments of Family and Community Medicine and Psychiatry and the Dalla Lana School of Public Health, University of Toronto, Toronto, ON Canada; 3 Ontario Tobacco Research Unit, Toronto, ON Canada; 4 Department of Family Medicine, Dalhousie University, Halifax, NS, Canada; 5 Cardiac Rehabilitation and Secondary Prevention Program, Toronto Rehabilitation Institute, Toronto, ON Canada; 6 Department of Medicine, University of Toronto, Toronto, ON Canada; 7 Health Economics & Outcomes Research, Pfizer Canada Inc, Kirkland, Québec, Canada; 8 Therapeutic Areas, Cardiovascular and Respiratory, Medical Division, Pfizer Canada Inc, Kirkland, Québec, Canada

**Keywords:** Clinical trial, Explanatory, Pragmatic, Smoking cessation, PRECIS, Varenicline, Bupropion, Nicotine replacement therapy

## Abstract

**Background:**

Numerous explanatory randomized trials support the efficacy of chronic disease interventions, including smoking cessation treatments. However, there is often inadequate adoption of these interventions for various reasons, one being the limitation of generalizability of the explanatory studies in real-world settings. Randomized controlled trials can be rated as more explanatory versus pragmatic along 10 dimensions. Pragmatic randomized clinical trials generate more realistic estimates of effectiveness with greater relevance to clinical practice and for health resource allocation decisions. However, there is no clear method to scale each dimension during the trial design phase to ensure that the design matches the intended purpose of the study.

**Methods:**

We designed a pragmatic, randomized, controlled study to maximize external validity by addressing several barriers to smoking cessation therapy in ambulatory care. We analyzed our design and methods using the recently published ‘Pragmatic–Explanatory Continuum Indicatory Summary (PRECIS)’ tool, a qualitative method to assess trial design across 10 domains. We added a 20-point numerical rating scale and a modified Delphi process to improve consensus in rating these domains.

**Results:**

After two rounds of review, there was consensus on all 10 domains of study design. No single domain was scored as either fully pragmatic or fully explanatory; but overall, the study scored high on pragmatism.

**Conclusions:**

This addition to the PRECIS tool may assist other trial designers working with interdisciplinary co-investigators to rate their study design while building consensus.

## Background

Schwartz and Lellouch [1] first used the terms ‘pragmatic’ to describe trials designed to help choose between options for therapy, and ‘explanatory’ to describe trials designed to test causal research hypotheses – for example, whether a particular intervention causes a specific biological effect. Randomized, double-blind, placebo-controlled trials that are largely explanatory are necessary to establish the safety and efficacy of new interventions and to inform evidence-based guidelines [2]. However, explanatory trials for chronic diseases such as hypertension, diabetes, depression and addiction have a number of limitations [3,4]. They are often conducted in tertiary centres, exclude people with comorbid conditions that cluster with the condition of interest, provide some incentive for participation, and mandate intensive follow-up visits and contact with research staff [5].

Explanatory studies, although highly internally valid, are often less generalizable to outpatient community settings. There has been a call for more real-world ‘practical’ or ‘pragmatic’ studies to enhance generalizability, but only rudimentary methods for distinguishing between efficacy (explanatory) and effectiveness (pragmatic) studies have been employed [1,3,4,6-8]. The definition and design of pragmatic trials vary considerably, and are derived mainly from descriptive papers. These often describe observational studies that, in spite of limitations in internal validity and the ability to control for confounders, have frequently been used to influence clinical practice [9,10]. Health policy makers have to make resource allocation decisions based on cost-effectiveness studies that may have excluded various populations of interest [3,11]. Therefore, randomized clinical trials with inherent internal validity, but with greater ecological and external validity – pragmatic, randomized trials – are required in real-world settings after safety and efficacy have been established.

**Figure 1:**
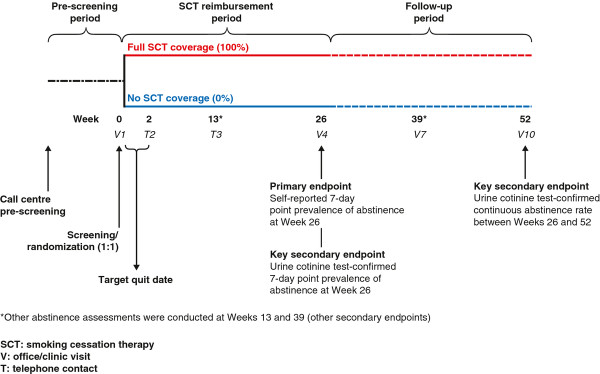
ACCESSATION study design.

In Canada, there is limited drug plan coverage for smoking cessation treatments despite their proven efficacy [12-15], ostensibly due to the lack of pragmatic, randomized trials. The multicentre, community-based, pragmatic, randomized, controlled ACCESSATION Study (ClinicalTrials.gov Identifier NCT00818207) was designed to determine whether smoking cessation treatment insurance coverage is associated with improved outcomes in clinical practice. We addressed two key barriers to smoking cessation treatment: i) the lack of formulary coverage for smoking cessation treatment by most governments and private drug plans in Canada (excepting the province of Quebec at the time of the study), and ii) the cost of medications to patients. Based on existing study design elements that favoured a pragmatic study design, we developed the study protocol to make the design as pragmatic as possible. The basic study flow (Figure 1) resembles that of a traditional randomized, controlled trial. However, for each aspect of the trial, we attempted to simulate real-world conditions; this was based on discussion among the authors.

After our study was initiated, an international consortium published the PRECIS (Pragmatic–Explanatory Continuum Indicator Summary) [16] model to help trialists assess the degree to which a study design falls along the pragmatic–explanatory continuum. The tool uses 10 key domains that qualitatively distinguish pragmatic (externally valid) from explanatory (internally valid) trials [16]. Although it has not been validated to predict outcomes *post facto*, no other instrument existed at the time and the consortium that developed this tool invites validation and enhancements to the process. Other limitations included the absence of a quantitative rating system that would increase precision, reproducibility and comparability of scores and of a formal process to reach consensus among investigators.

This paper describes the use of the PRECIS tool, coupled with the use of a numerical scale and a modified Delphi technique, to achieve consensus on the trial design, to characterize aspects of the study that determine whether it could be described as pragmatic.

## Methods

The 50 participating sites of the ACCESSATION Study that could utilize a Central Ethics Committee were reviewed and approved by Institutional Review Board (IRB) Services (Suite 300, 372 Hollandview Trail, Aurora, ON L4G 0A5, Canada), and the seven remaining sites submitted to an IRB that reviewed and approved the study in the respective regions.

We analysed the ACCESSATION Study trial design elements on a continuum using the qualitative, multidimensional PRECIS tool. Table 1 provides an overview of the most important study design characteristics in relation to the 10 domains of the PRECIS tool, and was developed by the primary author.

**Table 1:** Pragmatic and explanatory approaches in the ACCESSATION study

**PRECIS domain**	**Favours pragmatic trial**	**Criteria that do not favour either**	**Favours explanatory trial**	**Summary**
**Participants**				
Eligibility criteria	**·** Adult smokers (aged 18–75 years)**·** Prospective subjects were motivated to make a quit attempt within 14 days**·** Subjects were recruited from community-based sites across Canada**·** Comorbid patients included**·** Past compliance not considered**·** No risk stratification	**·** Smokers excluded if they have existing SCT; female subjects of childbearing potential could be included if not pregnant or nursing, and practising effective contraception (not eligible for within-label pharmacotherapy due to safety reasons)	**·** Smokers excluded if they had a quit attempt in the past 30 days, had unstable comorbid conditions, possessed SCTs, lived with another study participant or were currently participating in another study**·** Participants smoked ≥10 cigarettes per day**·** Eligibility had to be determined by a central service	**·** Mostly pragmatic
**Intervention and expertise**				
Experimental intervention – flexibility	**·** Physicians and patients controlled the type of smoking cessation interventions according to usual practice Any on-label combination of non- and pharmacological intervention was permitted**·** Participants could switch medications**·** Type or number of co-interventions used was not limited**·** Drug reimbursement card allowed any pharmacist to fill the prescription, rather than the doctor’s office**·** Side effects were to be managed at the discretion of the provider**·** Fewer contact minutes than efficacy trials with allowance for as-needed visits to assist the smoker	**·** Following randomization, a participant could not be re-randomized to a more favourable arm	**·** Provision of the card by the practitioner is not how insurance coverage cards are provided to patients**·** x`More contact than usual practice with scheduled rather than as-needed visits	**·** Mostly pragmatic
Experimental intervention – practitioner expertise	****·**** All physicians were licensed to practice in Canada, and included GPs/FPs (86%) and specialists in cardiovascular medicine, surgery, cardiology, pulmonology and psychiatry**·** Most investigators (80%) had no prior clinical research experience**·** Primarily treatment- seeking patients from physician practice (93%)**·** None of the authors were associated with sites of recruitment		**·** Some investigators (20%) had prior clinical research experience	**·** Mostly pragmatic
Comparison intervention – flexibility	**·** Participants received counselling about smoking cessation options and chose their SCT prior to randomization. This reflects usual practice		**·** Participants were given a drug reimbursement card, which included $5 to partially offset pharmacist dispensing fees, to enable tracking of SCT use if obtained from a pharmacy	**·** Mostly pragmatic
Comparison intervention – practitioner expertise	**·** Randomization was at the patient level to mimic the real world, so that within the same practice some smokers received reimbursement while others did not. Practitioner expertise was therefore the same for both arms at each site, reflecting the real-world variation in expertise in smoking cessation**·** Given the central randomization process, re-randomization to a more favourable arm (i.e., SCT coverage) was not possible		**·** Some training of smoking cessation occurred at the investigator meeting to ensure all practitioners had knowledge of evidence-based interventions for smoking cessation	**·** Mostly pragmatic
**Follow-up and outcomes**				
Follow-up intensity	**·** Participants did not attend the clinic on a weekly basis**·** At clinic visits/telephone contact, outcomes were measured, and AEs and method(s) used to quit (if any) were recorded**·** No exhaled CO levels were measured to reflect real-world practice		**·** Randomization visit involved more procedures than would usually be included at the first visit to discuss quitting**·** Although participants attended fewer contact visits than a traditional explanatory trial, they received more frequent contact than usual in clinical practice to balance the needs of data collection with the clinical reality of access to care**·** Strong focus on measuring outcomes and AEs from SCT use and/or quitting**·** A suicide severity scale was included because of recent reports of possible correlation with the use of SCTs	**·** Favours explanatory
Primary trial outcome	**·** Primary endpoint: comparison of the self-reported seven-day point prevalence of abstinence between the full and no reimbursement groups at Week 26 as opposed to 4 weeks continuous abstinence in efficacy trials in smoking cessation from the quit date. This endpoint is used in observational studies in telephone quitline and NRT distribution studies**·** Outcome assessed locally and no central adjudication of outcome		**·** Scheduled time for assessment of outcome at 26 weeks	**·** Balances both explanatory and pragmatic elements
Secondary endpoint	**·** A key secondary endpoint was self-reported 7-day point prevalence of abstinence at Week 26, confirmed by urine cotinine analysis. Urine collection is common in practice and the least intrusive method to confirm self-reports**·** Exhaled CO was not used, since this is less real-world practice		**·** Urine cotinine confirmation was used as a secondary endpoint to independently confirm self-reported abstinence. However, results were not shared with the practice	**·** Balances both explanatory and pragmatic elements
**Compliance/adherence**				
Participant compliance with intervention	**·** Once randomized, participants could choose to use (or not) any currently available SCT**·** SCT use patterns were passively measured by drug reimbursement card use across both groups**·** Data regarding adherence were collected but not shared with prescribers		**·** All or a portion of the participants were compensated for costs incurred to visit the clinic. They could have been compensated between $25 and $50 for each on-site visit (four on-site visits at V1, V4, V7 and V10). There was variation between some sites with the total allocation between $100 and $200 to account for local factors	**·** Mostly pragmatic
Practitioner adherence to study protocol	**·** There was no measure of how practitioners provided SCT *per se* or developed their source documentation for the study**·** There were some deviations from protocol, including prescribing off-label, no smoking cessation counselling before unblinding, and enrolling participants who were not motivated to quit		**·** Sites and physicians were visited by the site monitor twice during the study but kept to a minimum**·** Randomization was centrally controlled to prevent gaming. However, participants could use any available SCT**·** Standard study auditing processes discovered one site was not compliant with GCP (inadequate source documentation)**·** Study monitors would bring off-label use of medication to the attention of the investigator	**·** Balances both pragmatic versus explanatory trials
**Analysis**				
Analysis of primary outcome	**·** The main analysis will be the ITT population post-randomization regardless of use of the drug reimbursement card or making a quit attempt, with missing data being counted as being a smoker			**·** Mostly pragmatic

AE: adverse event; CO: carbon monoxide; GCP: good clinical practice; GP: general practitioner; FP: family practitioner; ITT: intent-to-treat; NRT: nicotine replacement therapy; SCT: smoking cessation treatment.

### Domain rating process

**Figure 2:**
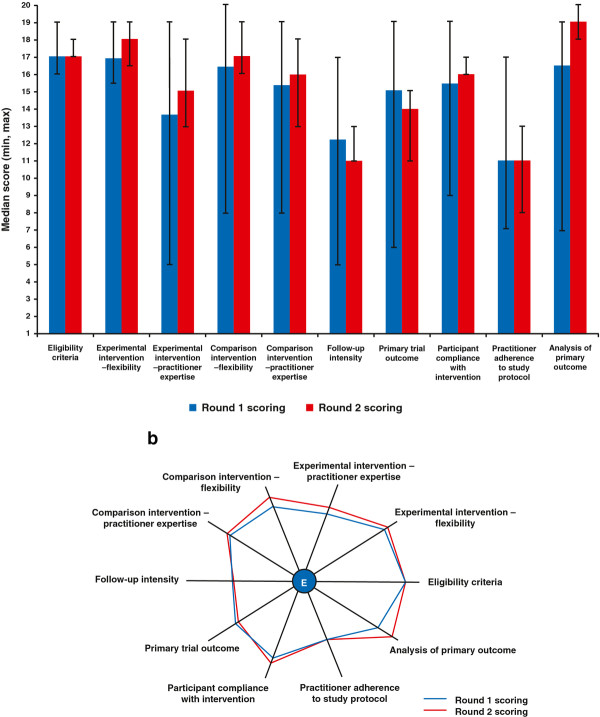
A. Author ratings for the ACCESSATION trial: median scores (min, max). B. Author ratings for the ACCESSATION trial using the pragmatic–explanatory continuum indicator summary (PRECIS).

To simplify the rating process, we added a quantitative aspect to evaluations using the PRECIS tool by adding a visual 20-point numerical scale, where 1 represented ‘entirely explanatory’ and 20 represented ‘entirely pragmatic’. Six raters (five authors and one consultant: one academic family physician with an interest in smoking cessation; one cardiac rehabilitation physician with expertise in pharmacoeconomics; one addiction medicine physician and clinical scientist with a focus on tobacco dependence; one pharmacist with expertise in pharmacoeconomics; one pharmacologist with clinical research and medical affairs experience in the pharmaceutical industry; and one consultant physician with pharmacoeconomic and policy advice experience in Quebec) were requested to score the trial on each domain in Table 1 according to where they believed it fell on a pragmatic–explanatory trial continuum, and to provide an explanation for their decision. Raters were asked to review Table 1 and to read the manuscript: ‘A pragmatic–explanatory continuum indicator summary (PRECIS): a tool to help trial designers’ [16] as a guide to scoring the 10 domains of the ACCESSATION Study. A modified Delphi technique was then used to ensure a common understanding among the raters, given their multidisciplinary background (addiction medicine, family medicine, internal medicine, pharmacy, pharmacoeconomics):

1. Initial group discussion among the raters regarding the study protocol and various elements classified by the 10 domains and criteria to justify their position, on a scale of 1 to 20 along the explanatory–pragmatic continuum.

2. Round 1 scoring: raters independently scored each domain (see domain rating process below); an independent assistant collated the scores and developed descriptive statistics (median, mean, standard deviation [SD]). The results were anonymized before the face-to-face group discussion.

3. A second face-to-face group discussion among the raters was held to clarify individual ratings, gain a better understanding of each domain and reach consensus.

4. Round 2 scoring: raters did a second round of rating independently and submitted their scores. Scores were again collated in an anonymized manner to generate descriptive statistics (Figure 2A). The results of the first and second rounds of scoring were plotted in a spider graph as described by the PRECIS tool developers [16] (Figure 2B).

## Results

Table 1 results, developed prior to the group discussions and domain rating process, suggested that only the domain related to ‘Follow-up intensity’ was considered more explanatory than pragmatic. Domains related to ‘Primary trial outcome’, ‘Secondary trial outcome’ and ‘Practitioner adherence to study protocol’ were considered to have a balance of both pragmatic and explanatory elements. However, the descriptive statistics calculated after ratings and discussions had taken place (Figure 2A) indicated that all 10 domains scored higher than the midpoint of 10.5. This indicates that all the domains were more pragmatic than explanatory – albeit some were borderline. Descriptive statistics (Figure 2A) indicated that there was less variation in scores after the second round than after the first round of discussions for every domain. This suggests that the raters’ opinions converged, presumably as a result of reaching a common understanding of all aspects of the trial in relation to each of the PRECIS domains. The spider plot (Figure 2B) demonstrates the shift in opinions among the raters between the first to the second round of discussions, with the plot becoming larger – more pragmatic – after the second round of discussions.

## Discussion

This paper describes the use of the PRECIS tool for the multidimensional evaluation of the ACCESSATION Study, and provides a thorough exploration of the study design that impacts its pragmatic/explanatory nature. Use of the tool highlighted study design features for which discrepancy of opinion existed among the authors regarding the degree of pragmatism within the trial, and provided a basis for discussing those areas more explicitly. However, this occurred after the study was initiated, but before data collection was completed. The high variability in ratings at the first scoring round was primarily due to differences in interpretation of the criteria described in the PRECIS tool and of how the design elements of the study fitted these dimensions. The dimensions were discussed and ratings were clarified based on the PRECIS tool. Therefore, it appears that deliberate discussion about each dimension is necessary, especially when there is considerable variability between raters. Use of a Delphi method is appropriate to reach consensus on such complex and subjective material.

If we were to design the study to be more pragmatic, we would reduce the frequency of visits for assessments and use a patient- and physician-defined primary outcome measure. For example, we would ask patients if they had quit or not, as opposed to using a validated scale. To make the study completely pragmatic on the primary outcome measure, we would use an administrative database to see if there was reduction in healthcare utilization in those who received coverage versus the control group.

Developers of the PRECIS tool [16] considered it to be an initial attempt to identify and quantify trial characteristics that distinguish between pragmatic and explanatory trials, and requested suggestions for its further development. Since 2010, five papers describing modifications to the PRECIS tool have been published, all of which employ quantification of the ratings on each dimension [17-21]. Each paper is summarized in Table 2.

**Table 2:** Studies attempting to quantify PRECIS dimensions since 2010

**#**	**Study**	**Author**	**Year**	**Ref.**	**Context of use and development**	**Numeric scale**	**Delphi/iterative process**	**Conclusions**	**Limitations**
1	The Pragmatic–Explanatory Continuum Indicator Summary (PRECIS) instrument was useful for refining a randomized trial design: experiences from an investigative team	Riddle et al.	2010	[17]	**·**Prospective use of the tool to modify study design in 3-arm study of pain coping in patients scheduled to have a knee replacement 1-day meeting of 7 authors/investigators: 1) Pre-read Thorpe et al., 20092) Discuss goal of study3) Discuss criteria4) Initial blinded rating5) Ideal blinded rating6) 3rd rating to see if consensus was reached	**·** Yes: 4-cm line to be marked independently on paper in person in 5 min followed by discussion **·** Scores measured by a ruler	Yes	**·** Modification of study**·** Closer consensus and more explanatory in design	**·** 4-cm scale using VAS; not applicable to online ratings
2	Alternative approaches to tuberculosis treatment evaluation: the role of pragmatic trials	Bratton et al.	2011	[18]	**·** Retrospective**·** 2 reviewers rated 3 published studies on TB treatment and discussed how to rate them on each dimension	**·** No, but modification to the dimensions made (practitioner expertise) combined and a new spoke for blindness inserted	**·** Yes, but not described in detail	**·** Pragmatic trials in TB might lessen time to implementation in real-world settings	**·** No metric at all, simply subjective
3	Pragmatic vs explanatory trials: the Pragmascope tool to help measure differences in protocols of mental health RCTs	Tosh et al.	2011	[19]	**·** Adapt (PRECIS) to assist researchers during protocol stage of RCTs in mental health (the Pragmascope tool)**·** Retrospective**·** 3 reviewers **·** Cochrane Schizophrenia Group Trials Register and Medline (November 2010) for references of RCT protocols. Chose a random sample of 10 protocols dealing with schizophrenia, depression, post-traumatic stress disorders, and psychiatric rehabilitation	**·** 1 to 5; 0 for missing information**·** Total score, 0 to 50**·** 0 to 30, explanatory; 31- 39, intermediate; ≥35, explanatory**·** However: Figure 1 (see main text) demonstrates an explanatory study investigating whether the experimental intervention will work in ideal circumstances (total score 0–15) and a more pragmatic study focusing mostly on whether, in routine practice, an intervention has a meaningful effect (total score >35)**·** A total score between 16 and 35 suggests an interim where trial design balances pragmatic and explanatory domains	**·** Not described	**·** Useful tool given high inter-rater reliability	**·** Scoring not clear; neither why the midpoint of 25 was not chosen as a balanced trial**·** Does the total score perpetuate the dichotomous classification of studies?
4	Applying the PRECIS criteria to describe 3 effectiveness trials of weight loss in obese patients with comorbid conditions.	Glasgow et al.	2011	[20]	**·** Practice-based Opportunities for WEight Reduction (POWER) trials (3 studies)**·** Reduce weight in primary care in those with one CVD risk factor. Studies were ongoing and in the field**·** 9 reviewers scored each protocol in a 4-step process. 1) Read article and review webinar by Sackett2) Score using 0 to 4 on the dimension**·** 8 additional ratings; representation of participants and settings, inclusion of cost estimates, reporting on context and level of engagement with the primary care practices	**·** 0 to 4 on the dimension with total composite scores also	**·** Not formally described, but does mention frequent calls to gain consistency in the interpretation of the dimensions	**·** Requires discussion and training to clarify each criteria	**·** ? Rater bias towards pragmatic**·** Scale difficult to interpret, but need cognitive testing as part of a qualitative study**·** Hard to find a reliability index
5	Pragmatic vs. explanatory: an adaptation of the PRECIS tool helps to judge the applicability of systematic reviews for daily practice	Koppenaal et al.	2011	[21]	**·** Improve lifestyle in general; should be implemented in general practice**·** Modified the PRECIS tool (called PRECIS Review tool [PR tool]) to grade individual trials and systematic reviews of trials**·** This should help policy makers, clinicians, researchers, and guideline developers to judge the applicability of individual trials and systematic reviews**·** 2 systematic reviews	**·** 1 to 5 rating**·** Individual studies and the review itself were scored**·** Abandoned VAS with 0 to 10 due to the arbitrary distinctions between consecutive scores**·** Used a Likert-type scale of 1 to 5, with concurrent %**·** If 3 dimensions unscorable, a randomized controlled trial was not to be used**·** 1 to 5 less arbitrary than 0 to 10 for this type of review	**·** 2 independent scores per study and then discussion to reach consensus**·** If unable, then consultation with third rater	**·**Useful tool for reviewers and policy makers and trialists**·** Ability to detect heterogeneity in studies included in systematic reviews	**·** Missing information more likely for a dimension to be rated as pragmatic**·** Applicability to setting and context needs to considered by policy makers even if a trial is considered pragmatic**·** Equal weighting for each dimension

RCT: randomized controlled trial.

In a similar analysis to ours, Riddle et al. used the PRECIS tool to design a randomized, controlled trial of pain-coping skills [17]. The authors also used the PRECIS tool to assist with face-to-face meetings and found the approach helpful, for similar reasons. They, too, added a semi-quantitative scale, but of 4 cm in length, and had three rounds of discussions. Their final evaluation led to greater agreement on all dimensions, whereby they increased the explanatory scores of each domain. The timing of their exercise prompted the authors to make revisions to the design of their randomized trial prior to submission for funding [17].

Tosh et al. [19] had three reviewers (co-authors) use a 1- to 5-point scale to review published trials in mental health; they referred to this as the Pragmascope. If a dimension could not be rated, it received a score of 0. Each trial could be allocated a total possible score of 50, with a range of 0 to 30 indicating an explanatory trial, 31 to 39 indicating a balanced trial and any score >35 indicating a pragmatic trial. However, in Figures 2 and 3, Tosh et al use ranges of 0 to 16 to describe explanatory trials and 16 to 35 to describe an interim trial that is balanced. They had independent ratings and averaging of scores, but did not describe an explicit process to be used to reach consensus.

Several limitations are associated with the use of the Pragmascope at this time. For example, if the dimension could not be rated, the dimension would receive a score of 0 and as such, bias ratings towards the study being explanatory. Moreover, the use of cut-offs for the total scores categorizing trials reverts to the problem of looking at trial design as purely explanatory or pragmatic [8,16]. Moreover, the reason for the cut-offs used is not specified. It is not clear why they did not choose 25 (the midpoint) to indicate a balanced trial and any score less than that would favour an explanatory study, while any score greater than 25 would favour a pragmatic study. Moreover, we agree with Glasgow et al. [5] and Spigt and Kotz [22] that composite scores should be avoided because widely disparate trials can receive the same score and defeat the purpose of having a dimensional approach to the rating.

The PRECIS Review (PR) tool was developed by Koppenaal et al. [21] to evaluate systematic reviews and the randomized controlled trials used in the review to help policy makers decide on applicable trials to inform their work. Like us, they quickly realised that a Visual Analog Scale (VAS) scale of 0 to 10 was arbitrary and so converted it to a Likert-type scale of 1 to 5, also including a percentage score. They used two reviewers and an additional reviewer to rate the score when consensus could not be reached. The scoring scale appeared to be valid for the stated purpose and they acknowledged the limitations of broader applicability. Again, given the purpose behind the PRECIS tool to introduce multidimensionality to the evaluation of a study design, scores are important to initiate and guide discussion, but broader consensus on the rating is still required to inform decision making.

Glasgow et al. [20] also used a 5-point (0 to 4) scale to rate three interrelated, yet separate studies by investigators from three separate institutions. They describe a similar process of training reviewers and noted that investigators tended to rate their own papers as being pragmatic. The scoring revealed moderate levels of variability with most variability within 1 point on the 5-point scale. However, several telephone calls were required to develop consensus on the meaning of each score. It is possible that the scale was not sufficiently sensitive to detect a difference, which would be important if the group was interested in achieving consensus, but less so if trying to evaluate the study *per se* and categorize the protocol dichotomously.

Our proposed refinement also identified the need for a rating scale, but included a modified Delphi technique to reach consensus [23]. We chose a 20-point numerical scale to approximate a continuous scale. This permitted easier, more accurate and more stable coding of the response using e-mail. A VAS with measurement is appropriate when standardized in pen and paper format rather than e-mail, which distorts the dimensions. We also used extreme anchor points, 1 to 20, to discourage rating the domains beyond the numbers provided. Moreover, Likert scales have increased reliability with up to 11 steps, 7 steps being the minimum. Therefore, the scale we used was most sensitive to capture inter-individual differences to better target our discussions. This may be one reason why the spider graphs do not reach the extremes, but it is also possible that the raters appreciated that elements existed in each dimension to prevent an extreme rating. Use of the iterative technique provided a sound basis for discussing the intricacies of the trial design and allowed individuals to provide viewpoints anonymously and then offer their opinions during face-to-face meetings.

Taken together, these examples demonstrate that depending on the purpose of the application of the PRECIS tool (study evaluation versus study design), different scales and methods may need to be used to rate studies. However, our method may be particularly helpful to trialists to ensure common understanding of a study design when working in teams with disparate expertise. Therefore, other investigative teams may find these approaches helpful.

The multidimensional PRECIS tool can be implemented easily by investigators and represents a major advance in the design and evaluation of clinical trials that inform practice, as demonstrated by our own experience and that of others. All clinical trialists need to make compromises in their design due to a variety of practical factors that affect the conduct of a large study. Collaborative research by a team requires consensus on study design to ensure the methods are appropriate to answer the study question. Methods to evaluate study design and reach consensus are needed to ensure that disparate views and perspectives can be reconciled so that the best possible course of action is adopted.

Although most agree that the 10 dimensions are necessary to understand the explanatory–pragmatic continuum, numerical scales run the risk of dichotomously classifying the study and we did not provide a composite score for the study. This required a qualitative approach. Therefore, a more structured process using the Delphi technique that we employed, or a similar nominal group technique used by Riddle et al. [17], allowed a more democratic process of consensus among the investigators, who hailed from different disciplines and institutions. This process may be helpful to investigators during the design stage of a multicentre collaborative study to resolve disagreements and assist in reaching a common understanding of the design of the study.

## Conclusions

The PRECIS tool may be applicable across a variety of health-related studies to help investigators design trials most appropriate to their study question and hypothesis. Moreover, clinicians, study reviewers, policy makers and the so-called post-regulatory decision-makers can use this tool to determine if a study has generalizability to the populations of interest and the level of reasonable effectiveness that can be expected in different ecological settings versus those in explanatory trials. In these situations, simpler rating systems as described by others might be adequate to achieve the desired outcome.

## Abbreviations

IRB: Institutional Review Board; PR: PRECIS Review; PRECIS: Pragmatic–Explanatory Continuum Indicatory Summary; SD: Standard deviation; VAS: Visual Analog Scale.

## Competing interests

All authors have completed the Unified Competing Interest form at http://www.icmje.org/coi_disclosure.pdf (available on request from the corresponding author). All non-Pfizer authors declare financial compensation from Pfizer Inc. for professional services, including protocol and clinical trial materials development, initial start-up and end-of-study activities such as Case Report Form review, Statistical Analysis Plan review, preparation, participation and presentation at the Investigator Meeting, and Clinical Study Report review. No payments were made by Pfizer Inc. to PS, GB or PO for authorship and/or authorship-related activities of this paper. Travel expenses such as airfare, hotel accommodation, meals and ground transportation were reimbursed by Pfizer Inc. when appropriate. During the conduct of the study, PS and GB received research funding from their respective employers.

## Authors’ contributions

PS conceptualized the idea for the paper, the addition of the Delphi Panel and the semi-quantitative scale for each dimension. He also prepared Table 1 for discussion and prepared the first draft. All authors participated in and made a substantial contribution to the discussions of the conception and design, and acquisition analysis and interpretation of data. They also revised the article critically for intellectual content and gave final approval of the version submitted for publication. All authors read and approved the final manuscript.

## Pre-publication history

The pre-publication history for this paper can be accessed here:

http://www.biomedcentral.com/1471-2288/12/101/prepub
